# “A randomized, double-blind study of the effects of omega-3 fatty acids (Omegaven™) on outcome after major liver resection”

**DOI:** 10.1186/s12876-015-0331-1

**Published:** 2015-08-14

**Authors:** Michael Linecker, Perparim Limani, Florin Botea, Irinel Popescu, Ruslan Alikhanov, Michail Efanov, Pavel Kim, Igor Khatkov, Dimitri Aristotele Raptis, Christoph Tschuor, Beatrice Beck-Schimmer, John Bonvini, Andrea Wirsching, Philipp Kron, Ksenija Slankamenac, Bostjan Humar, Rolf Graf, Henrik Petrowsky, Pierre-Alain Clavien

**Affiliations:** 1 Swiss Hepato-Pancreato-Biliary (HPB) and Transplantation Center, University Hospital Zurich, Zurich, Switzerland; 2 Department of Surgery & Transplantation, University Hospital Zurich, Raemistrasse 100, CH-8091 Zurich, Switzerland; 3 Department of Anesthesiology, University Hospital Zurich, Zurich, Switzerland; 4 Center of General Surgery and Liver Transplantation, Fundeni Institute Bucharest, Bucharest, Romania; 5 Department of Liver and Pancreatic Surgery, Moscow Clinical Scientific Center, Moscow, Russia

**Keywords:** Omega3- polyunsaturated fatty acids (n3-PUFA), Omegaven™, Major liver surgery, Clavien-Dindo complication score, Comprehensive Complication Index (CCI)

## Abstract

**Background:**

The body is dependent on the exogenous supply of omega-3 polyunsaturated fatty acids (n3-PUFA). These essential fatty acids are key players in regulating metabolic signaling but also exert anti-inflammatory and anti-carcinogenic properties. The liver is a major metabolic organ involved in fatty acid metabolism. Under experimental conditions, n3-PUFA exert beneficial effect on hepatic steatosis, regeneration and inflammatory insults such as ischemic injury after surgery. Some of these effects have also been observed in human subjects. However, it is unclear whether perioperative administration of n3-PUFA is sufficient to protect the liver from ischemic injury. Therefore, we designed a randomized controlled trial (RCT) assessing n3-PUFA (pre-) conditioning strategies in patients scheduled for liver surgery.

**Methods/Design:**

The Omegaven™ trial is a multi-centric, double-blind, randomized, placebo- controlled trial applying two single doses of Omegaven™ or placebo on 258 patients undergoing major liver resection. Primary endpoints are morbidity and mortality one month after hospital discharge, defined by the Clavien- Dindo classification of surgical complications (Ann Surg 240(2):205–13, 2004) as well as the Comprehensive Complication Index (CCI) (Ann Surg 258(1):1–7, 2013). Secondary outcome variables include length of Intensive Care Unit (ICU) and hospital stay, postoperative liver function tests, fatty acid and eicosanoid concentration, inflammatory markers in serum and in liver tissue. An interim analysis is scheduled after the first 30 patients per randomization group.

**Discussion:**

Long-term administration of n3-PUFA have a beneficial effect on metabolism and hepatic injury. Patients often require surgery without much delay, thus long-term n3-PUFA uptake is not possible. Also, lack of compliance may lead to incomplete n3-PUFA substitution. Hence, perioperative Omegaven™ may provide an easy and controllable way to ensure hepaative application of tic protection.

**Trial registration:**

ClinicalTrial.gov: ID: NCT01884948, registered June 14, 2013; Institution Ethical Board Approval: KEK-ZH-Nr. 2010–0038; Swissmedic Notification: 2012DR3215.

## Background

Before the 1930s, dietary fat was thought to be simply a source of calories which could be exchanged by e.g. carbohydrates. In 1930, Mr. & Mrs. Burr discovered that specific fatty acids were critical to health [3]. Linoleic acid was the first essential fatty acid described to restore growth deficits and prevent dermatitis in rats given fat-free diets [3].

**Table 1:** Study synopsis

Sponsor investigator	Prof. Pierre-Alain Clavien, MD PhD
Study product	Omegaven™, 100 ml intravenously administered at the evening before and during liver resection
Primary endpoint	Postoperative morbidity and mortality determined by the Clavien- Dindo classification of surgical complications and the Comperative Complication Index (CCI) 1 month after hospital discharge.
Secondary endpoints	Main center:
	Serum samples: postoperative peak AST and ALT, fatty acids and n3 PUVA concentration, inflammatory markers
	Liver biopsy: histology (necrosis, apoptosis), inflammatory markers, hepatic fatty acid and n3 PUVA content
	Main center and external centers:
	Duration of hospitalization and ICU stay
	Hematology: hemoglobin, hematocrit, leukocytes, platelets, INR
	Chemistry: triglycerides, bilirubin, AST, ALT, ALKP, creatinin, CRP
Methodology	Randomized, double-blind, placebo controlled
Clinical phase	Phase III (new indication for Omegaven™)
Study duration	3 years (start: July 2013)
Study centers	Multi-center (Zurich, Bucharest, Moscow)
Number of subjects	258 patients
Main inclusion criteria	Adult (more than 18 years) requiring liver resection of at least 1 segment or multiple wedge resections (≥3); no coagulopathy (INR ≤ 1.2, platelets ≥ 150,000 × 10^3^/μl)
Main exclusion criteria	Liver resections <1 segment, wedge resections (<3); coagulopathy (INR > 1.2, platelets < 150,000 × 10^3^/μl); hypertriglyceridemia (>5.0 mmol/l); liver cirrhosis; severe renal failure (estimated GFR < 30 ml/min/1.73 m^2^); pregnancy.
ClinicalTrial.gov	ID: NCT01884948

**Fig. 1:**
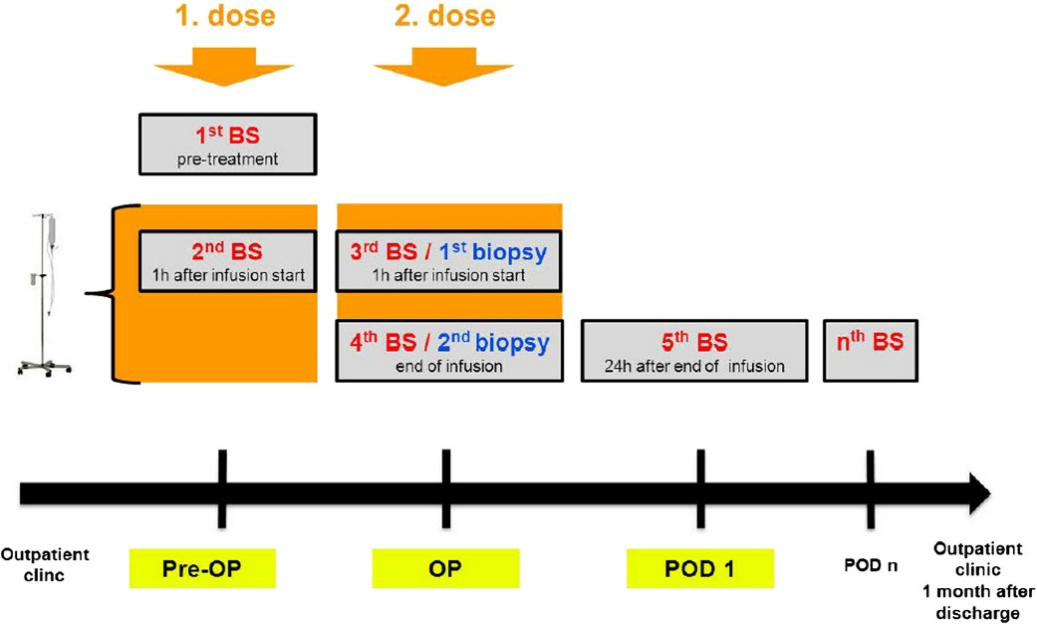
Study flow chart overlooking the study period schematically. (BS: blood sample, OP: Day of operation, POD: Post- operative day)

**Fig. 2:**
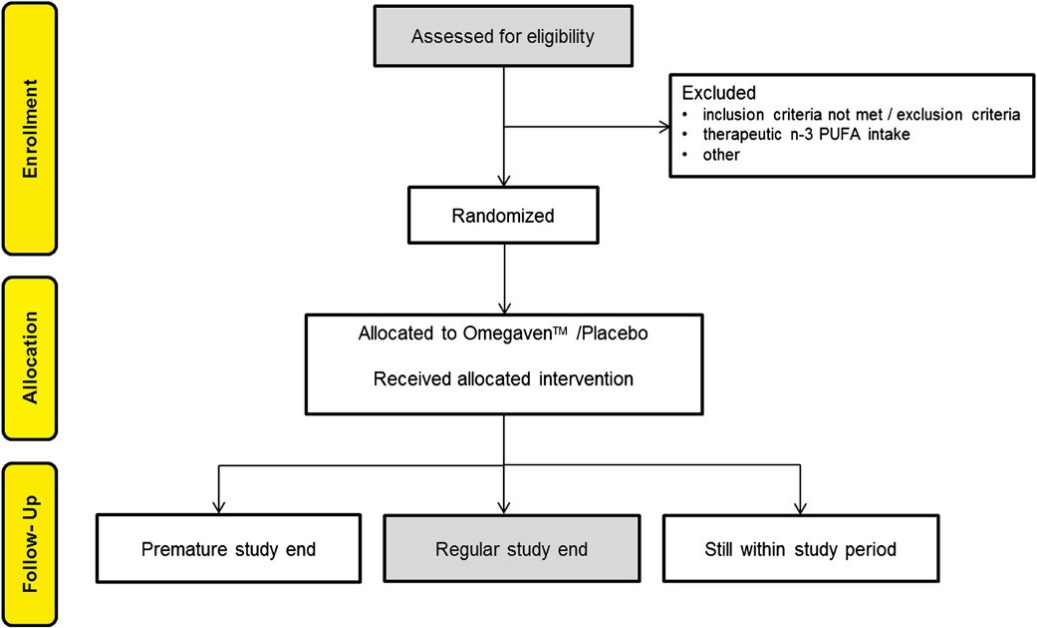
Study accountability according to the CONSORT statement [11]

In the late 1970s Bang & Dyerberg have associated the exceptionally low prevalence of coronary heart disease among Greenland inuits with their high intake of omega-3 polyunsaturated fatty acids (n3-PUFA) from marine sources [4]. To date, after more than 40 years of intense research, an abundance of beneficial effects have been attributed to n3-PUFA.

Undoubtedly, the liver is a central target of n3-PUFA given that this organ maintains systemic metabolic control. Preclinical data suggest that supplementation of n3-PUFAs may be beneficial in operations involving steatotic livers [5], after major liver resection and subsequent regeneration [6], and during inflammatory insults such as ischemic injury [5, 7, 8]. Moreover, some beneficial effects have been documented also in human subjects [9]. The protective effect of n3-PUFA supplementation is being primarily investigated in a long term setting, which seems not very appropriate for patients scheduled for liver resection. First, surgery should not be delayed to reach a comparable duration of n3-PUFA oral administration. Second, intravenous perioperative administration renders an easy and controllable way of protection and circumvents problems of compliance.

Major liver surgery can incur serious postoperative complications, with ischemic injury and regeneration being two major determinants of the operative outcome. Fatty liver additionally increases postoperative complications, as it inhibits regeneration and displays explicit sensitivity towards ischemic injury. More so, fatty liver can progress to steatohepatitis and hepatocellular carcinoma, a major indication for liver surgery. Thus, steatosis indirectly increases the need for surgery whilst limiting its application. Perioperative treatment with n3-PUFA, on the other hand, may reduce steatosis, mitigate ischemic injury and promote regeneration, effects likely of great benefit for fatty liver. Therefore, n3-PUFA are expected to lower the incidence and/or the level of complications following liver surgery.

In this trial, we will assess the use of n3-PUFA as a potential (pre-)conditioning strategy for the reduction of postoperative complications after major liver surgery. Omegaven™ is the first n3-PUFA formulation approved for the intravenous use in humans. In the patient collective scheduled for liver resection, parenteral administration seems more appropriate for reasons mentioned above. Owing to the favorable toxicity profile, the ease of application, and the general health-promoting effects, (pre-)conditioning through n3-PUFA has the potential to become a standard strategy to improve the outcome of major liver surgery (Table 1).

## Methods/Design

The Omegaven™ trial is a prospective, multi-centric, double-blind, randomized, placebo- controlled trial assessing the effect of two peri-operative doses of Omegaven™ (100 ml each dose) on morbidity and mortality after major liver resection. The trial is open to all high-volume hepato-biliary centers performing at least 30 cases/year potentially eligible for this study. So far, three centers are recruiting patients. Ethics approval for the main center Zurich was obtained from Professor Edith Schmid, for Bucharest from Professor Mihai Voiculescu and for Moscow from Professor Byakhov M.Y. Additional centers are open to start recruiting after local regulatory approval. In total 258 patients will be recruited and an interim analysis performed when 30 patients from each group have successfully reached the primary study endpoint.

### Study objectives

The study aims at a 30 % reduction in the Comprehensive Complication Index (CCI) or a 30 % reduction of any complication according to the Clavien- Dindo score [1]. Secondary endpoints include duration of hospitalization and Intensive Care Unit (ICU) stay, readmission rates, postoperative peak ALT (alanine aminotransferase) and AST (aspartate aminotransferase) serum levels (area under the curve), fatty acid and n3-PUFA concentration in serum and liver tissue as well as assessment of inflammation in liver biopsies. N3-PUFA concentrations in sera and studies on liver biopsies will be measured and performed in the main center only.

### Inclusion criteria

- Requiring liver resection of at least one segment or multiple wedge resections (≥3)
- > 18 years of age
- Understand local language
- No coagulopathy (international normalized ratio (INR) < 1.2, platelets >150,000 × 103/μl)

### Exclusion criteria

- Liver resections <1 segment
- Wedge resections (<3)
- Liver cirrhosis on histology
- Coagulopathy (INR > 1.2, platelets < 150,000 × 10^3^/μl)
- Hypertriglyceridemia (>5.0 mmol/l)
- Hypersensitivity or allergy to Omegaven™ or any fish oil or lipid emulsions
- Known allergy to egg protein
- Pregnancy
- Nursing women
- Renal failure (estimated GFR < 30 ml/min/1.73 m2)
- Medication impairing platelet aggregation *Study product*

Omegaven™ is a fish oil-based lipid emulsion approved for parenteral nutrition provided by Fresenius Kabi (Swissmedic approval number: 54750). Both study drug and placebo (0.9 % saline) are administered in 100 ml equally shaped bottles with a maximum infusion rate of 0.5 ml/kg/hour on the evening before surgery and during surgery.

### Blinding

In this double-blind RCT surgeons and nurses are blinded. Since the placebo is transparent and Omegaven™ is a white emulsion, blinding is done with a black bag covering the study medication along with opaque infusion lines. Blinding is performed by a study-independent person, which is also responsible for randomization. This person is not involved in the study otherwise. Before intravenous administration, this person has to ensure that blinding is done properly and the patients and study personnel cannot detect the color of the infusion. An emergency code break will be available to the investigator. This code break should be opened only in situations where the identity of the investigational product must be known by the investigator in order to provide appropriate medical treatment.

### Randomization

A block randomization is performed by the CTC (Clinical Trial Center) Zurich to minimize a potential center bias. In detail, the above-mentioned independent person (i.e. responsible for the blinding) of each center receives a block randomization list which is adjusted to the case load of the center. As soon as a patient is included, a “randomization- button” in the electronic case report form (eCRF) will allocate the patient to the randomization-number in the block randomization list.

### Recruitment, study procedures and data collection

Patients potentially eligible for the study are first seen in the outpatient clinic where demographic data and medical history are evaluated and physical examination is performed. Eligible patients (according to the inclusion/exclusion criteria) will be asked whether they are willing to participate in the study. Those willing to participate will be asked to provide a written informed consent. Patients having given written consent will be further evaluated for eligibility. The preoperative workup will be performed according to local practice. Routine laboratory tests as well as the mentioned laboratory parameters will be determined (including Serum TG, INR and platelet count). Women will undergo a pregnancy test. If serum measurements are within a normal range and female patients are not pregnant, randomization can be performed the day before surgery. Subsequently, the first infusion of Omegaven™ or Placebo is administered. One hour after infusion start, safety blood samples assessing circulating TG levels, platelets and INR are taken. Major deviations from baseline levels along with clinical symptoms will terminate study drug application. The second dose starts the following morning during induction of anesthesia in the operating theatre. As done during the first administration, safety blood samples are obtained one hour after infusion start followed by another blood sample at the end of infusion. Liver biopsies and blood samples for the determination of inflammatory markers and n3-PUFA concentrations are assigned to the main center only. The last perioperative blood sample is taken 24 hours after finishing the administration of the second dose. Further blood samples rely on routine clinical practice of the participating centers. According to the definition of the Clavien Dindo score [1] of surgical complications and the CCI [2], patients will reach the primary study endpoint one month after hospital discharge. All data including adverse and serious adverse events (AE and SAE) will be prospectively collected using a software specifically designed for the purpose and needs of this study (secuTrial™). SAE from all centers are reported to the Sponsor- Investigator of the trial who is responsible for further reporting to the regulatory authorities (IEC and Swissmedic) (Figs. 1 and 2).

### Sample size

We hypothesized that in 117 patients two intravenous infusions of Omegaven™ will protect the liver causing effective reduction of the post-operative complication rates (CCI) by 30 % when compared to the 117 placebo patients.

The sample size of 117 patients per randomized group (234 in total) was calculated based on the following parameters using the patient cohort of major liver resections at the University Hospital Zurich:

- Percentage of complications for placebo group: 65 %
- Mean CCI in major resections is 17 (standard deviation: 15.9)
- Percentage of complications for Omegaven™ group:
  30 % reduction of complications and/or CCI
- α: 0.05
- Power (1-β): 0.8
- 2-sided test

Expected differences in proportions are based on data derived from the literature. Such a statistically significant reduction of the complication rate is considered to be clinically significant. A 10 % dropout rate is relatively common for liver resection, we thus have increased the sample size to 258 patients in total for both groups.

### Ethics

The study is conducted in accordance with principles enunciated in the current version of the Declaration of Helsinki, the guidelines of Good Clinical Practice (GCP) issued by the International Conference on Harmonization (ICH), and Swiss regulatory authority’s requirements. The study protocol, patient information and consent form are approved by the Independent Ethics Committee (IEC) in agreement with local legal requirements at each study site. Any amendments to the protocol, other than administrative ones, must also be approved by these committees.

## Discussion

The goal of this RCT is to assess for major liver surgery the hepato-protective effects of two perioperative infusions of an n3-PUFA preparation. This type of surgery is inherently associated with a relatively high complication rate pending on the complexity of the procedure. Therefore, pharmacological strategies to protect the remnant liver during this traumaperiod are in the focus of interest.

The major point of interest is if two single peri-operative doses will suffice to induce the postulated protective effect. A recent kinetic study in healthy volunteers revealed that a single oral dose of n-3 PUFA is able to rapidly induce a shift in the n-3 PUFA plasma profile within a few hours [10]. Also, short term administration just prior to ischemic insults was strongly protective in animal models [7]. These findings support our hypothesis that one dose of n3-PUFA as pre-conditioning (the day before surgery) followed by another continuous infusion during liver surgery elevates n3-PUFA plasma levels to potentially therapeutic levels.

The study medication, Omegaven™ is approved by Swissmedic since years for the indication of parenteral nutrition and is thus the n3-PUFA preparation of choice for intravenous use. In this trial, the best comparative placebo would contain the same intravenous emulsion without n-3 PUFA. However, from a pharmaceutical point of view such a placebo is not feasible, as it would require its own clinical safety assessment. Hence, saline was chosen.

Given the liver-specific defatting effect combined with protective effects for surgical trauma (i.e. mitigation of ischemia- reperfusion injury and improvement of regeneration) n3-PUFA appear to be a promising and feasible strategy for safer liver surgery.

## Abbreviations

AE: Adverse event
ALT: Alanine transaminase
AST: Aspartate transaminase
CTC: Clinical trials center
eCRF: Electronic case report from
GCP: Good clinical practice
GFR: Glomerular filtration rate
ICH: International conference on harmonization
ICU: Intensive care unit
IEC: Independent ethics committee
IIT: Investigator initiated trial
INR: International normalized ratio
n3-PUFA: Omega3- polyunsaturated fatty acids
RCT: Randomized controlled trial
SAE: Serious adverse event
TG: Triglycerides
